# Heart and skeletal muscle inflammation (HSMI) disease diagnosed on a British Columbia salmon farm through a longitudinal farm study

**DOI:** 10.1371/journal.pone.0171471

**Published:** 2017-02-22

**Authors:** Emiliano Di Cicco, Hugh W. Ferguson, Angela D. Schulze, Karia H. Kaukinen, Shaorong Li, Raphaël Vanderstichel, Øystein Wessel, Espen Rimstad, Ian A. Gardner, K. Larry Hammell, Kristina M. Miller

**Affiliations:** 1 Pacific Biological Station, Fisheries and Oceans Canada, Nanaimo, British Columbia, Canada; 2 Pacific Salmon Foundation, Vancouver, British Columbia, Canada; 3 School of Veterinary Medicine, St. George's University, Grenada, W. Indies; 4 Centre for Veterinary Epidemiological Research, Department of Health Management, Atlantic Veterinary College, University of Prince Edward Island, Charlottetown, Prince Edward Island, Canada; 5 Department of Food Safety and Infection Biology, Norwegian University of Life Sciences, Oslo, Norway; INRA, FRANCE

## Abstract

Heart and skeletal muscle inflammation (HSMI) is an emerging disease of marine-farmed Atlantic Salmon (*Salmo salar*), first recognized in 1999 in Norway, and later also reported in Scotland and Chile. We undertook a longitudinal study involving health evaluation over an entire marine production cycle on one salmon farm in British Columbia (Canada). In previous production cycles at this farm site and others in the vicinity, cardiac lesions not linked to a specific infectious agent or disease were identified. Histologic assessments of both live and moribund fish samples collected at the farm during the longitudinal study documented at the population level the development, peak, and recovery phases of HSMI. The fish underwent histopathological evaluation of all tissues, Twort’s Gram staining, immunohistochemistry, and molecular quantification in heart tissue of 44 agents known or suspected to cause disease in salmon. Our analysis showed evidence of HSMI histopathological lesions over an 11-month timespan, with the prevalence of lesions peaking at 80–100% in sampled fish, despite mild clinical signs with no associated elevation in mortalities reported at the farm level. Diffuse mononuclear inflammation and myodegeneration, consistent with HSMI, was the predominant histologic observation in affected heart and skeletal muscle. Infective agent monitoring identified three agents at high prevalence in salmon heart tissue, including *Piscine orthoreovirus* (PRV), and parasites *Paranucleospora theridion* and *Kudoa thyrsites*. However, PRV alone was statistically correlated with the occurrence and severity of histopathological lesions in the heart. Immunohistochemical staining further localized PRV throughout HSMI development, with the virus found mainly within red blood cells in early cases, moving into the cardiomyocytes within or, more often, on the periphery of the inflammatory reaction during the peak disease, and reducing to low or undetectable levels later in the production cycle. This study represents the first longitudinal assessment of HSMI in a salmon farm in British Columbia, providing new insights on the pathogenesis of the disease.

## Introduction

Heart and skeletal muscle inflammation (HSMI) in Atlantic Salmon was reported for the first time in Norway in 1999 [1]. Since then, the number of HSMI outbreaks has steadily increased to a peak of 181 cases reported in Norway in 2014 [2]. Although HSMI is not notifiable to the World Organisation for Animal Health (OIE), HSMI is considered to be the third most important salmon disease in Norway [3]. The disease has been reported also in Scotland [4] and Chile [5,6].

The current diagnostic criteria for HSMI are based on histopathologic lesions in the heart and skeletal muscles that distinguish this disease from other known diseases in salmon [7]. In the heart, moderate to severe myocarditis (in both compact and spongy layer) is characterized by infiltration of mononuclear inflammatory cells (primarily lymphocytes and macrophages). The epicardium is also strongly affected by an inflammatory reaction, while the atrium is less involved; the latter may show lesions similar to those occurring in the spongy layer of the ventricular myocardium, but are usually milder [8,9]. Along with the inflammatory pattern, myodegeneration and necrosis can be present. The skeletal muscle also shows moderate to severe myodegeneration and necrosis occurring in the red muscle fibres only as well as inflammatory infiltration [1]. However, lesions in the heart are more frequently observed when fish are in a compromised disease state (initial stage of the disease, peak of the disease and recovery phase), while the skeletal muscle is generally impacted during peak occurrence of HSMI lesions and, to a lesser extent, in the recovery phase [10,11]. For this reason, a tentative histopathological diagnosis of HSMI at the individual fish level requires at least the presence of described heart lesions. Cardiac lesions in the later stage of HSMI, however, can overlap the pattern of lesions seen in other diseases, like Cardiomyopathy Syndrome (CMS), and hence the lesions in the red fibers of skeletal muscle act as a differentiating element [1,11]. Other histopathological lesions concurrently found are generally considered the result of circulatory disturbance due to heart failure, like multifocal zonal or patchy liver necrosis and congestion or hemorrhages occurring in liver, kidney, spleen and gills, while the pancreas remains unaffected by any inflammatory or degenerative reaction [1].

Additional features characteristic of the disease are a low mortality, from negligible up to 20%, and high morbidity, up to 100% of the farm population [1]. Outbreaks often occur 5–9 months after transfer to seawater, although the disease has been reported to occur also in freshwater [12,13]. Clinically-affected fish can show abnormal swimming behaviour and anorexia, and ascites, pale heart and liver, splenomegaly, and visceral petechiae are the typical gross lesions reported [1,9]. Nevertheless, increased mortality and clinical signs are not reliable indicators for the extent of an outbreak [11]. Moreover, unlike outbreaks of salmonid Pancreas Disease (PD), which also affects the heart and where up to 15% of the survivors can fail to grow [14], “poor performers” have not been commonly associated with HSMI [9].

Although HSMI in Atlantic Salmon has been reported with concurrent presence of *Piscine orthoreovirus* (PRV) and has not been reproduced in the absence of PRV [15–17], to date, a causal relationship between the virus and disease has not been demonstrated. Questions on PRV’s role in the development of HSMI stem from the relatively ubiquitous nature of the virus in farmed populations [18,19], high qPCR loads of PRV occasionally found in apparently healthy fish (farmed and wild) with no HSMI lesions [19–21], and lack of success in culturing the virus in continuous cell lines [12,22]. Moreover, PRV has been reported in fish presenting CMS concurrently with *Piscine Myocarditis Virus* (PMCV) [23] in farmed Atlantic Salmon, where PRV was considered acting as an “opportunistic” pathogen. A further study also demonstrated the concurrent presence of PRV with Salmonid Alphavirus (SAV) in farmed Atlantic Salmon [24]. However, two studies attempting to sequence any agent with a RNA genome related to cell culture passage preparation or tissues from two different HSMI outbreaks in Norway, only revealed PRV [17,25]. A third study identified a novel calicivirus (*Atlantic salmon calicivirus*—ASCV) associated with HSMI fish; a challenge study using pure ASCV resulted in lesions consistent with HSMI, but it was later determined that these fish also had PRV [16]. Analysis of field outbreaks of HSMI found no correlation between ASCV and HSMI [24].

While myocarditis lesions have been reported sporadically across multiple farms in British Columbia (BC) dating as far back as 2002, no particular disease, including HSMI, has been diagnosed in association with these lesions. Our study involved longitudinal sampling of apparently healthy and moribund fish through a marine production cycle to evaluate temporal changes in prevalence of observed histopathologic lesions and how they may relate to multiple agents associated with diseases in salmon worldwide. In a parallel study by our group, assessing associations between histopathologic lesions and agents in regulatory audit samples collected throughout the industry, we observed four farm cases of myocarditis lesions between 2011 and 2013 in the region where we had collected the longitudinal farm samples in 2013–2014. Careful examination of dying fish from these four farms through the regulatory Fish Health and Surveillance program (Department of Fisheries and Oceans—Aquaculture Management Division) revealed lesions consistent with HSMI (S1 File). The study herein documents the analyses that were then undertaken to characterize the pathological development of these lesions. The samples included moribund (or recently dead when no moribund fish were available) and apparently healthy salmon collected on the farm over the entire ocean production cycle and were assessed by histopathology, immunohistochemistry, molecular monitoring of 44 infectious agents, and on-site clinical data. Throughout, we use the case definition for the diagnosis of HSMI outlined by Biering and Garseth [7] that is applied broadly across Norway, which bases the diagnosis of the disease strictly on the occurrence of pathological lesions. However, we incorporated by association clinical signs, mortalities, and pathogen data that, while not used specifically in the diagnosis of HSMI, were used to compare with published reports of HSMI outbreaks in Norway. By applying a longitudinal study design, we document the first diagnosis of HSMI on a farm in British Columbia, which was evident over an 11-month period and was highly consistent with similar outbreaks on Norwegian farms.

## Materials and methods

### Case definition of HSMI

In this study, we followed the diagnostic case definition used by Biering and Garseth [7], wherein the diagnosis of HSMI is based on histological inflammatory lesions occurring in heart and skeletal muscle, but not in the pancreas. However, while inflammatory lesions in the skeletal muscle may indicate HSMI at the population-level, and in particular can help to differentiate HSMI from CMS, skeletal muscle lesions are not consistently present throughout an entire HSMI outbreak, and hence are not always a useful differential at the level of the individual fish [9–11]. Details of the histological classification of the heart and skeletal muscle lesions are reported in the Histopathology section. We did not use any other inclusion criteria (e.g. mortality, concurrent clinical signs, or molecular results with specific cut-off values) to modify our case definition for HSMI disease, as these criteria have been previously considered to be unreliable indicators throughout an entire HSMI outbreak [11,18].

### Sampling protocol

#### Ethics statement

All work with animals was performed in strict accordance with the recommendations in the Canadian Council on Animal Care (CCAC) Guide to the Care and Use of Experimental Animals. The protocols were approved by the DFO Pacific Region Animal Care Committee (Animal Use Protocol Number: 13–008). Euthanasia on live and moribund fish was performed by overdose of tricaine methanesulfonate (Syndel laboratories Ltd., Nanaimo BC, Canada).

#### Fish and tissue collection

All tissue samples collected for this program were collected under a Material Transfer Agreement between the BC Salmon Farmers Association and Fisheries and Oceans Canada. The twelve pens of the farm analysed in this study were fully stocked in May 2013, and after a one-month acclimation, sampled bi-monthly for the first 5 months, with collections of 30 random samples of live “clinically healthy” fish and up to 10 moribund and/or freshly dead fish (where possible), followed by monthly samples of 20 live and up to 12 moribund/recently dead fish until harvest began 18 months later. Different pens (chosen by the company veterinarian) were sampled at each sampling date to even out the handling stress among pens. Sample dates used in this study focussed the analysis on the temporal period surrounding the peak of histopathological lesions in the heart, and included 75% of the entire collection, as depicted in Table 1. Details on the tests performed on each sample are showed on S2 File.

**Table 1:** Summary of the samples analyzed.

Sampling date	Fish sampled			Test performed					
	Live	Moribund	Dead	Histo	BioMark^™^	Stat	HC	IHC(PRV Sigma-1)	IHC(*K*. *thyrsites*)
19/06/2013	30	4	0	34					
03/07/2013	0	7	5	12					
14/08/2013	30	1	2	33(33)					
28/08/2013	30	2	10	42	42	37	10	2	5
11/09/2013	30	3	8	41(10)	41	38	5	5(3)	3
25/09/2013	30	1	8	39				7	
09/10/2013	30	1	10	41				10(1)	
23/10/2013	30	0	9	39	39	37		13	2
05/11/2013	30	9	4	43(43)	43	41	7	22(8)	3
04/12/2013	20	5	0	25				7	
15/01/2014	20	5	0	25(13)	25	25		5(5)	
12/02/2014	20	1	3	24					
09/04/2014	20	1	6	27					
04/06/2014	17	3	2	22					
10/09/2014	20	2	0	22					
Total	357	45	67	469(99)	190	178	22	71(17)	13

The table shows the number of samples undergoing histopathological evaluation (Histo), microfluidics-based qPCR screening for 44 agents (listed in S3 File) (BioMark^™^), Statistical evaluation associating agents with observed lesions (Stat), Twort’s Gram staining (HC) and immunohistochemistry against two antigens (IHC—PRV Sigma-1 and IHC—K. thyrsites). The brackets in the “Histo” column indicate the samples read by both pathologists. Failed samples for histological or molecular analysis (no housekeeping gene or single Ct values) were excluded from the Statistical evaluation. The brackets in the “IHC (PRV Sigma-1)” column indicate the samples tested also with a second PRV antibody (PRV Mu1c).

All fish were euthanized with overdose of tricaine methanesulfonate (250mg/l; Aqualife TMS, Syndel laboratories Ltd., Nanaimo BC, Canada) and dissections were conducted on-site using aseptic technique. Dissections provided six tissues for molecular testing, including brain, liver, head kidney, heart, muscle and gill, although only heart tissue was included in this study for molecular testing; the tissues were preserved individually in RNAlater, held at 4°C overnight, then stored at -80°C degrees until homogenization. Tissues for histopathological investigations were collected from the same fish and preserved in 10% neutral buffered formalin; tissues included gills, heart, brain, eye, liver, anterior and posterior kidney, spleen, stomach, pyloric caeca including pancreas and skin at the lateral line to include red and white muscle. Blood samples were also collected, transported to the laboratory where they were centrifuged and frozen (-20°C) as soon as possible (6–24 hrs post-collection), for use in future studies.

#### Clinical findings and mortality data

During the field sampling events, the veterinarian involved in this study took general and specific notes reflecting observations including clinical signs and gross lesions for all moribund/dead fish, but not for live fish sampled, while farm-level observations were provided by farm technicians. All findings were transferred electronically into an Excel spreadsheet, capturing their original descriptions. Briefly, the dataset consisted of columns for each description of clinical sign or gross lesion (n = 82), and the cells were populated with fish totals for moribund and recently dead fish for each farm sampling event. The descriptions of clinical signs and lesions were later aggregated to include five broad categories for: sea lice, mouth rot disease (MMY; myxobacteriosis), poor performers (PP), central nervous system (CNS; e.g. inflammation, hemorrhage, etc.), and HSMI-related. For the latter category, any of the following were included: slow-swimmer, off-feed, ascites (e.g. ascites, hemorrhagic ascites, visceral petechiae, etc.), liver (e.g. enlarged, congested, etc.; excluding “green” and “haemorrhagic”), spleen (e.g. enlarged, congested, etc.), and heart (e.g. enlarged, pericardial effusion, etc.). Although these findings have all been reported to be present concurrently to HSMI outbreaks in Norway, they do not represent pathognomonic signs or lesions of the disease, as they can be reported for other conditions too.

Daily mortality data were provided, by the Company owning the farm in this study, for every pen throughout the production cycle. The mortality counts were aggregated at the farm-level and summarized into weekly records for each of the following six categories recorded as the causes associated with mortalities: handling, predatory attack (e.g. otters), MMY, skin- and fin-related lesions, environmental (i.e. harmful algal bloom and low dissolved oxygen levels), and other (all remaining mortality counts). Mortalities are shown as percentages calculated from the closing fish count from the previous week.

### Laboratory tests

#### Histopathology

All tissues fixed in 10% neutral buffered formalin were dehydrated through an ascending gradient of alcohol solutions, embedded in paraffin wax, cut at 3.5μm thickness and stained with standard hematoxylin and eosin (H&E) for morphological evaluation by light microscope.

H&E stained slides were prepared for all moribund fish collected across all four farms included in the project, and examined initially to gauge the range of lesions and diseases expected within the single farm samples. These histopathological examinations were then extended to include live (apparently healthy) and freshly dead fish on a single farm showing distinctive inflammatory lesions in heart and skeletal muscle tissues, with a total of 469 fish assessed over a 16-month period (June 2013 to September 2014) for that farm.

Upon diagnosis of HSMI at the farm, we considered that the other two major viral diseases representing differentials for HSMI (namely PD and CMS) and their respective agents are not known to be present in BC and were not present in our samples. Therefore, all fish with any degree of myocarditis consistent with HSMI were considered to be within the “HSMI complex” and classified according to the scoring system used by Finstad et al. [26]. Under this scoring regimen, lesions were classified in a range from 0 (no lesions) to 3 (most severe lesions) for each of the two heart components (epicardium and compact/spongy myocardium). Fish having a cumulative score from both components of 4 or higher were diagnosed as HSMI fish [27], and thereby represented fish with the most severe lesions. Fish with a cumulative score between 3 and 3.5 were considered as moderate lesions, and a score of 1.5 to 2.5 was indicative of mild lesions. Finally, fish scoring 1 or less were considered to be HSMI negative fish (no lesions). Previous challenge [26] and farm-level [11] studies have demonstrated that HSMI-diagnostic lesions in the heart move from the outside inward (i.e. from epicardium and small myocardial blood vessels to compact myocardium to spongy myocardium) over the course of the disease. We devised a scheme to further classify our fish with mild to moderate lesions as fish in a “developing stage” of HSMI or a “recovery stage”. The developmental pathway of the disease was therefore assessed by observing the presence, extent and position of the lesions in the heart and using the ratio between lesion severity scores in the epicardium versus the myocardium (which included the spongy layer). A ratio > 1 indicated that a fish was in the “developing stage” of the disease (more damage in the epicardium than the myocardium), while a ratio ≤1 was considered to be a “recovering stage” fish (damage moving to the interior of the heart).

Skeletal muscle lesions were also analysed and scored according to a semi-quantitative scoring system. In detail, lesions occurring in the red fibres were classified in a range from 0 to 3. A score equal to 0 designated no inflammatory reaction, while a score of 1 was assigned when the area involved in the inflammatory response represented less than 30% of the red fibers on the histology section. A score of 2 was assigned when the area involved comprised between 30 and 60%, and finally a score of 3 when more than 60% of the red fibres were involved in the inflammatory reaction. It is worth mentioning that previous studies did not use a scoring system to classify the lesions occurring in the skeletal muscle, other than categorizing them as no lesions, mild, moderate or severe.

Morbidity, defined in our study as the percentage of fish with heart scores ≥1.5 (i.e. fish with some degree of histopathological lesions in the heart consistent with HSMI, including all live and moribund fish sampled) was also assessed.

Two pathologists were involved in the diagnosis of disease. One pathologist read the histology slides from all 469 fish, and after initial analyses, focussed primarily on heart and skeletal muscle tissue. A second pathologist conducted a complete pathological analysis (blinded to the initial results) of all tissues on a sub-sample of 99 fish, covering the whole sampling period and including a mixture of fish initially deemed positive or negative to HSMI. Specifically, those sub-sampled included fish from before HSMI occurrence (August 14, 2013, n = 33), a random selection of samples from the beginning of the episode (September 11, 2013, n = 10), fish sampled during the peak phase at the farm level (November 5, 2013, n = 43) and a random selection of samples from the recovery stage (January 2014, n = 13). Agreement between the two pathologists was assessed using McNemar’s chi-square test and qualitative interpretation of kappa was based on Landis and Koch [28].

#### Histochemistry

A subset (n = 22, from the total of 469) of samples showing high load of the two most prevalent parasites in the heart (as determined by quantitative q-PCR described below) with and without concomitant heart lesions underwent Twort’s Gram staining, in order to localize the organisms in the tissues. In essence, we tested eight samples for *Kudoa thyrsites* and 14 samples for *Paranucleospora theridion* (syn. *Desmozoon lepeophtherii*). It is noteworthy to mention that the samples tested with this staining procedure included (1) specimens in which these parasites were the only organisms present in the heart as per our independent infective agent monitoring, (2) slides where both parasites were present, and (3) a random selection of samples during the peak phase of the HSMI occurrence and with both parasites present at high load.

#### Immunohistochemistry

Throughout the entire production cycle, sections from all fish with a histological score for heart lesions ≥4 (n = 56), sampled between June 2013 and January 2014, underwent immunohistochemistry staining using a polyclonal rabbit antibody against PRV Sigma-1 protein (Anti PRV σ1, #K275) [26]. This was done to determine whether PRV, detected by molecular screening of heart tissue, was localized in the affected area of the heart in a manner similar to that observed in farmed Norwegian salmon with HSMI. In addition, a selection of fish without histopathological lesions in the heart and negative for PRV (n = 4) and fish presenting high loads of PRV without histopathological lesions in the heart (n = 7) or mild lesions (n = 4) also underwent the same staining procedure to determine the localization of the virus in these samples. To further confirm the presence of PRV in the heart lesions in a second laboratory, a subset of fish (n = 17out of the 56) with score ≥4 were also stained with rabbit polyclonal antibody against PRV Mu1c protein (Anti PRV μ1c, #K265), accordingly to a previously-published protocol [26].

Fish showing high load of *K*. *thyrsites* in the heart (Ct ≤10, as determined by qPCR; n = 8 out of 469) and fish showing concomitant heart lesions (n = 5 out of 469) underwent IHC with mouse anti *K*. *thyrsites* monoclonal antibody (clone IPA-2F4, Thermo Scientific, Rockford CA, USA), in order to localize the parasite in the tissue and its possible association with the lesions.

For all the antibodies, 3.5 μm thick paraffin wax embedded sections were mounted on Superfrost Plus glass slides (Thermo Fisher Scientific, Portsmouth, USA). These were heated at 60°C for 20 min, dewaxed in xylene and rehydrated through graded alcohols. Antigen retrieval was performed by autoclave treatment (121°C for 10 min) in citrate buffer (0.1 M, pH 6.0). The sections were successively treated with 3% hydrogen peroxide for 1h to block endogenous peroxidase activity. Non-specific binding sites were blocked by normal goat serum (Vector Laboratories, Burlingame, CA, USA) diluted 1:10 in 1% bovine serum albumin (Vector Laboratories, Burlingame, CA, USA) in TBS [pH 7.6, 0.05 M Tris/HCl, 0.15 M NaCl] for 1h. The same diluent solution was used for primary antibodies (1:3000 for anti PRV Sigma-1 and Mu1c, as for Finstad et al. [26]; 1:20 for anti *K*. *thyrsites*, as per the manufacturer’s instructions) and then incubated in a humidity chamber at 4°C overnight. A Vectastain ABC-peroxidase kit (Vector Laboratories, Burlingame, CA, USA) was used for detection of bound antibody according to the manufacturer’s instructions, and ImmPACT NovaRED (Vector Laboratories, Burlingame, CA, USA) or DAB (Vector Laboratories, Burlingame, CA, USA) as substrate. Finally, the sections were counterstained with Harris haematoxylin and mounted (Permount). All incubations, except with the primary antibodies, were carried out at room temperature in a humidity chamber. PRV/HSMI positive (n = 2) and negative (n = 1) heart samples from Atlantic Salmon collected during an experimental challenge for HSMI [29] were used as positive and negative controls for anti PRV Sigma-1 and PRV Mu1c antibodies. Skeletal muscle samples from Atlantic Salmon seen by histological evaluation to be heavily infected with *K*. *thyrsites* and containing also heart tissue were used as positive controls (n = 2), while apparently healthy heart samples from Atlantic Salmon, negative to *K*. *thyrsites* by qPCR, were used as negative controls (n = 1). Primary and secondary antibody controls were performed by replacing the antibody with the respective diluent alone.

#### Infective agent monitoring

We employed the Fluidigm BioMark^™^ microfluidics-based qPCR system developed for salmon infective agent monitoring to quantitatively assess 44 agents (11 viruses, 11 bacteria, and 22 fungal and protozoan parasites) known or suspected to infect or be carried in salmon worldwide [30]. The complete list of all the agents tested is reported in S3 File.The analytical sensitivity and specificity, and repeatability of the system have been evaluated for these 44 agents [30]. Heart samples were selected based on a temporal window identified through histopathology, from June 2013 to January 2014, with 190 fish analysed in total.

Nucleic acid extraction was performed as follows: Homogenization using Tri-reagent^™^ was performed in a Mixer Mill (Qiagen, Maryland); homogenates were processed for RNA extraction using one of the three replicate aqueous layers and the Magmax^™^-96 for Microarrays RNA kit (Ambion Inc, Austin, TX, USA) with a Biomek NXP^™^ (Beckman-Coulter, Mississauga, ON, Canada) automated liquid-handling instrument, both based on manufacturer’s instructions. The quantity of RNA was analysed using spectrophotometer readings and normalized to 62.5ng/μl with a Biomek NXP (Beckman-Coulter, Mississauga, ON, Canada) automated liquid-handling instrument, based on manufacturer’s instructions. Heart RNA (1μg) was reverse transcribed into cDNA using the superscript VILO master mix kit (Invitrogen, Carlsbad, CA) following manufacturer’s instructions. The cDNA was then used as template for the Specific Target Amplification (STA) step.

While we were not multiplexing assays on the BioMark, the STA (run on a conventional PCR machine) did involve multiplexing low concentrations (1/20th of normal concentrations) of all primers to be run on a single dynamic array; these unincorporated primers were degraded in a purification step (Exo-SAP-IT^™^ has no interference in downstream application, 100% recovery of PCR products) before individual assays were run. The 5 μl STA reaction contained 1.3 μl of cDNA/DNA, 1X TaqMan PreAmp master mix (Applied Biosystems, Foster City, CA, USA) and 0.2 μM of each of the 47 primers (44 agents and 1 housekeeping gene assay from [30]. The STA cycling program was performed according to manufacturer’s instructions for TaqMan gene expression assays (Fluidigm Corporation, South San Francisco, CA, USA) and included 14 cycles [31].

Upon completion of the STA, excess primers were removed by treating with Exo-SAP-IT^™^ (Affymetrix, Santa Clara, CA) according to manufacturer’s instructions and then diluted 1/5 in DNA re-suspension buffer (Teknova, Hollister, CA). To ensure primers were completely removed, a mixed probe/no primers control was achieved by amplifying samples post Exo-Sap-It treatment.

The 96.96 gene expression dynamic array (Fluidigm Corporation, CA, USA) was run according to the procedure outlined in [30]. Specifically, a 5 μl template mixture was prepared for each sample containing 1 × TaqMan Universal Master Mix (No UNG), 1 × GE Sample Loading Reagent (Fluidigm PN 85000746) and each of diluted STA’d sample mixtures. Five μl of Assay mix was prepared with 1 × each of the appropriate TaqMan qPCR assays (agent probe in FAM-MGB and artificial construct [APC] probe in NED-MGB, 10 μM of primers and 3 μM of probes) and 1 × Assay Loading Reagent (Fluidigm PN 85000736).

Controls were added prior to running the dynamic array, as per [30]. Note APC clones to all assays were contained in a single serially diluted pool, loaded last, minimizing the likelihood of contamination of any single APC clone. Once loading and mixing of the dynamic array was completed within the IFC HX controller, the array was transferred to the BioMark HD instrument and processed using the GE 96x96 Standard TaqMan program for qPCR which includes a hot start followed by 40 cycles at 95°C for 15 sec and 60°C for 1 min (Fluidigm Corporation, CA, USA). The data were analysed with Real-Time PCR Analysis Software (Fluidigm Corporation, CA, USA). Processed data were uploaded to Access via Excel tables and analysed in GenEx.

#### PRV sequence

Two samples with high loads of PRV were selected for high throughput sequencing of RNA (dual RNA-seq) to resolve the full genome sequence of PRV. RNA-seq libraries were prepared using the ScriptSeq Complete Epidemiology NGS library kit (Illumina, San Diego, CA), barcoded, and combined into an RNA-seq run on the Illumina MiSeq platform (Illumina, San Diego, CA).

Total RNA extracted from heart tissue samples was evaluated for quality using the Total RNA Pico chip on the Agilent 2100 Bioanalyzer (Agilent, Santa Clara, CA) and quantity using the Qubit RNA Br kit (Invitrogen, Carlsbad, CA). A 1/100 dilution of the ERCC RNA Spike-In control mix 1 (Ambion, Carlsbad, CA) was added to each total RNA sample prior to ribosomal depletion and library preparation. The sequencing libraries and ribosomal removal were performed using the Epicentre ScriptSeq Complete Gold Kit (Epidemiology) (Illumina, San Diego, CA) according to manufacturer’s instructions and included a positive control (Universal Human Reference RNA) (Agilent, Santa Clara, CA) and negative control (no total RNA). The rRNA depleted total RNA was purified using the Zymo RNA Clean and Concentrate-5 kit (Zymo Research, Irvine, CA) according to manufacturer’s instructions and quantified using the Qubit RNA HS kit (Invitrogen, Carlsbad, CA). The ScriptSeq Index reverse primers were added to the cDNA during the final amplification step which involved 13 cycles. The 3’-terminal tagged cDNA and final amplified library were purified using the Agencourt AMPure XP system (Beckman Coulter, Brea, CA) at 1.8X and 1.0X ratios, respectively. The final library size was determined using the HS DNA chip on the Agilent 2100 Bioanalyzer (Agilent, Santa Clara, CA) and the concentration was determined using the Qubit dsDNA HS kit (Invitrogen, Carlsbad, CA). Sample libraries were normalized to 2-4nM, pooled appropriately and denatured and diluted to obtain a final library of 20pM. Prior to loading into a v3 2x300 bp kit (Illumina, San Diego, CA), 5% phiX was spiked in. Finally, a paired-end 301bp sequencing run was performed on the Illumina MiSeq System (Illumina, San Diego, CA).

#### PRV genome sequence analysis

Sequence analysis was performed using the Partek Flow software (Partek Inc. St. Louis, MO, USA). Adaptors and bases with Phred quality scores <30 were trimmed from both ends and reads less than 25bp were removed. The remaining reads were aligned to the PRV genome segments of the Norwegian isolate Salmo/GP-2010/NOR [25] using the BWA-mem aligner with default parameters. SAMtools and FreeBayes variant callers were utilized to determine SNPs using the default settings. Finally, consensus sequences were generated utilizing variant calls, chromosome visualizations through reference alignments and Sequencher 5.1 software (Gene Codes Corporation, Ann Arbour, MI). The consensus sequences were compared against all available sequences in Genbank [32] using the BLAST program via the National Center for Biotechnology Information [33] to identify their closest matches and mismatches across each segment. PRV segment consensus sequences for B5690 and B7274 isolates were deposited into Genbank under the accession number series KX851964 to KX851983.

### Statistical analysis

The main objective for our statistical analyses was to identify significant predictors associated with heart lesions. For the purpose of this study, only three agents associated with heart lesions (PRV, *K*. *thyrsites* and *P*. *theridion*) were included in the analyses described below as they were the only organisms exceeding 5% prevalence in the samples analysed. Data were recorded and handled using Excel, and all analyses were performed using STATA v14 (StataCorp, College Station, Texas, USA). Agent loads were normalized (transformed) by using a base 10 logarithmic scale [log10(copy number + 1)]. Ranges, means, medians, and standard errors were analysed for agent load over sampling dates, heart scores, condition of fish at time of sampling, and PRV prevalence. A 3-category heart lesion score was created from the histopathologists’ findings: HS0 = total heart score≤1, HS1 = total heart score >1 and <4, and HS2 = total heart score ≥4; these cutpoint values were based on the Finstad et al.’s semi-quantitative scoring system [26]. Seasons were classified as winter (December to February), spring (March to May), summer (June to August), and autumn (September to November). Time was analysed as five sampling dates between August 2013 and January 2014, and the ‘fish condition’ referred to whether or not the samples came from recently dead (D), moribund (M), or live (L) fish. To capture as much histological information as possible, an ordinal logistic regression was used to model the 3-category heart lesion score. The presence of PRV in the heart was included as a dichotomous predictor (0/1), and we also accounted for time (5 categories) and the fish condition (D, M, or L). All predictors were included as fixed effects, and potential interaction terms were included between PRV, sampling dates, and fish condition. Given the sparseness of data over time and across heart scores, we simplified the evaluation of statistical interaction of predictors and imposed assumptions during the model building process. As such, we limited our interactions to dates, simplified predictors (e.g. using dichotomous PRV instead of load or copy numbers) for parsimony, and assumed proportional odds across the histological scores.

## Results

### Descriptive epidemiology of the events occurring on the farm

**Fig 1:**
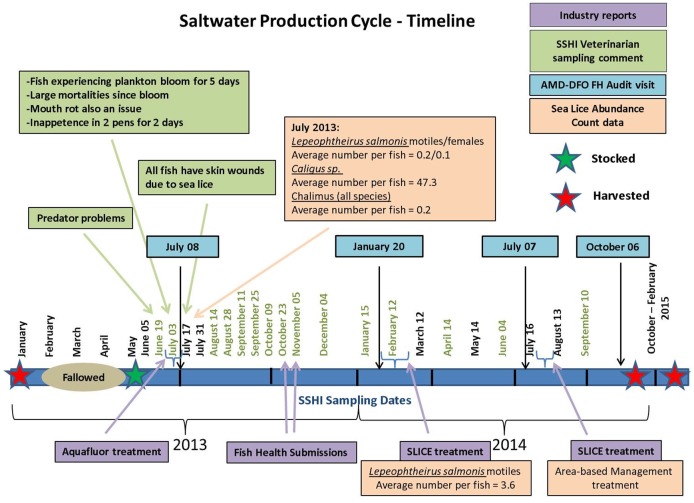
Timeline of the events occurring at the farm during the period of study. All sampling events carried out at the farm are shown. The Federal regulatory Fish Health and Surveillance audit dates carried out by the Aquaculture Management Division of Fisheries and Oceans Canada (AMD-DFO FH) are boxed in blue. Dates for Sea lice Abundance Count Data supplied by industry are boxed in peach (indicating average number of sea lice per fish, including a distinct count for *Lepeophtheirus salmonis* motiles and females, *Caligus sp*. and chalimus lifestage for all species, when concurrently present in the same fish). Industry reports of medical treatment and Fish Health submissions boxed in purple. Comments from the veterinarian hired for this study as part of the Strategic Salmon Health Initiative (SSHI) program boxed in green. The samples analysed in the study belong to sampling dates colored in green.

The timeline of events occurring at the farm is shown in Fig 1. Following a four-month fallowing period, the farm was fully stocked with approximately 50–55,000 fish per pen over twelve pens in May 2013 with Atlantic Salmon smolts originating from two different hatcheries at week 17–19 of production cycle (body weight > 100 g). However, one pen received salmon in early April from another ocean production site (but still coming from one of the same hatcheries) at week 6 (body weight > 90 g) after they were transferred into the ocean. Ten fish from each of the two hatcheries underwent testing for PRV prior to the ocean transfer; all fish tested negative. The company reported a harmful algal bloom event (*Heterosigma sp*.) occurring in late June 2013, lasting about 5 days, followed by mouth rot disease (treated with AQUAFLUOR^®^), associated with elevated mortality (>0.5% weekly mortalities for several weeks between June and July 2013) and suspension of feeding behaviour in affected fish. On July 17^th^ 2013, the veterinarian performing the sampling also reported the presence of wounds associated with sea lice in all the fish collected. In that date, average number of *Caligus sp*. per fish was reported to be 47.3, but the average number of *Lepeophtheirus salmonis* motiles per fish was only 0.2, therefore no mandatory management action was taken. Two laboratory submissions from the farm veterinarian were sent for further analysis in October 22^nd^ and November 5^th^, 2013. By February 2014, the count for *L*. *salmonis* motiles reached the average number of 3.6 per fish, prompting delousing treatment (SLICE^®^). A delousing treatment was also performed in late July 2014 as “preventive” area-based management treatment, due to nearby farms reaching the threshold of three *L*. *salmonis* motiles per fish. Fish were harvested between October 2014 and February 2015.

During the production cycle, four AMD-DFO Fish Health and Surveillance Audit visits were performed. These are undertaken by the regulatory branch on a random sample of farms from the approximately 40 to 50 production sites in BC.

### Clinical findings and mortality data

**Fig 2:**
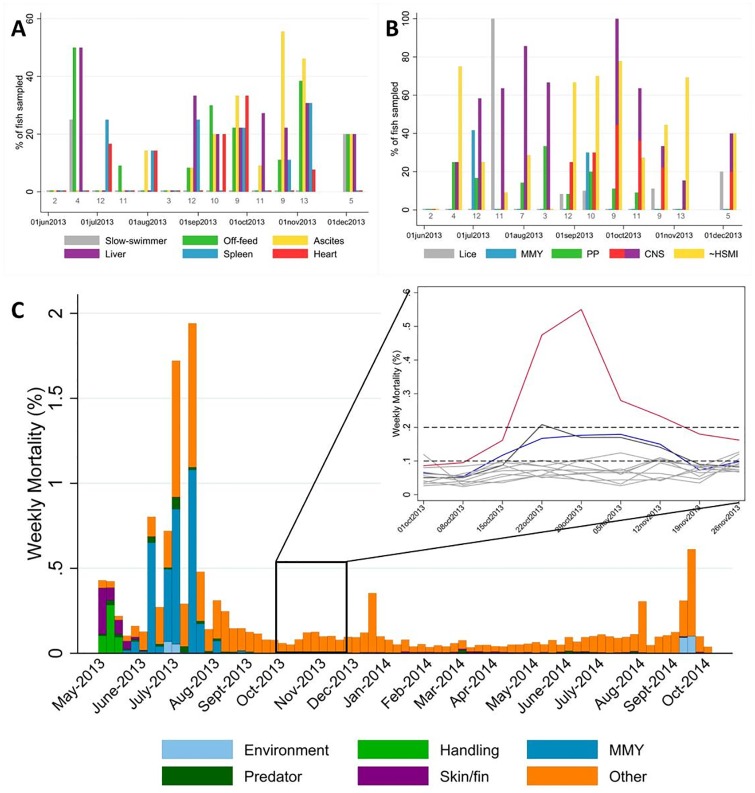
Clinical signs, gross lesions, and weekly mortalities reported for the farm in this study. A) Non-specific clinical signs and gross lesions which have previously been associated with HSMI, as reported by Kongtorp and colleagues [1,9,11]. Any of the following were included: slow-swimmer, off-feed, ascites (e.g. ascites, hemorrhagic ascites, visceral petechiae, etc.), liver (e.g. enlarged, congested, etc.; excluding “green” and “haemorrhagic”), spleen (e.g. enlarged, congested, etc.), and heart (e.g. enlarged, pericardial effusion, etc.). B) All clinical signs and lesions presented in “A” were further aggregated into one category, called “~HSMI”, and compared to additional lesions and clinical signs observed over the course of the production cycle. The latter were aggregated to include five broad categories for: sea lice, mouth rot disease (MMY; myxobacteriosis), poor performers (PP), central nervous system (CNS; e.g. inflammation in red, hemorrhages in purple), and HSMI-related (from Fig 2A). Percentages of fish sampled showing clinical signs or lesions were calculated from the number of fish collected (moribund or dead) at each sampling event (light grey font color located above the sampling date). C) Weekly mortality counts (provided by the company) summarized based on their general classification. The inset demonstrates weekly mortality for each pen of the farm between October 1^st^ and November 26^th^, 2013: during that period, one pen had increased mortalities (red line), with two more pens minorly affected (royal blue and charcoal lines), and the company veterinarian had submitted fish samples as part of routine investigation.

The clinical observations were available for 108 fish collected at the one farm included in this study. There were a total of 34 moribund and 74 recently dead fish, and a summary of their combined clinical signs is presented in Fig 2A and 2B. During the temporal period when HSMI lesions in the heart samples were present, clinical signs included being off feed and slow swimmers (Fig 2A). Gross lesions observed in the field included ascites (often haemorrhagic), brain haemorrhages and inflammation, kidney and spleen congestion/enlargement, pale and/or enlarged liver, pale and/or enlarged heart and pericardial haemorrhages (Fig 2A). In particular, ascites occurrence followed the development of HSMI, reaching a peak of 56% and 46% of moribund and dead fish in October and November 2013, respectively.

Based on the weekly-aggregated mortality data on the farm (Fig 2C), and observations recorded by the farm veterinarian, there were several events associated with increased mortalities, such as: the initial stress from handling and stocking pens (a 3-week period in May 2013); harmful algal bloom and mouth rot disease (8–10 weeks between June and August 2013); fluctuating dissolved oxygen and variable feed rates affecting predominantly one pen in October-November 2013, peaking at 0.55% (weekly mortality recorded during the week of Oct 29^th^, 2013), with two other pens only minorly affected, although farm-wide peak mortality in this period was <0.13%; an on-site power failure in mid-December (harsh weather conditions with snow, frozen equipment, and loss of winter lights); and intermittent episodes of low dissolved oxygen (<4.0 mg/l observed on slack tides, with no harmful algal blooms detected, in August, September, and October 2014).

### Histopathological lesions and developmental pathway of HSMI over time

**Fig 3:**
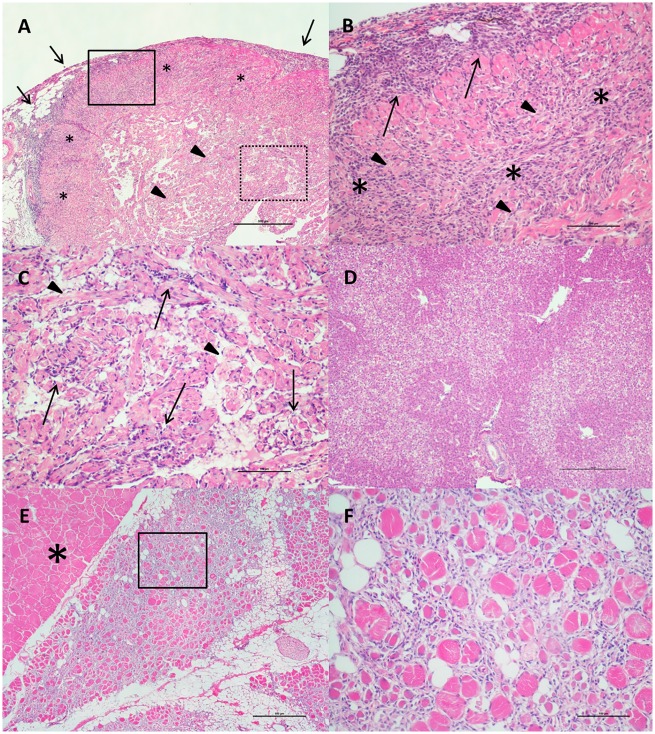
Heart and Skeletal Muscle Inflammation (HSMI) lesions in BC farmed Atlantic salmon (H&E). A) Severe diffuse/ infiltrative panmyocarditis, involving the epicardium (arrows) and the compact layer of the myocardium (stars). The spongy layer is also involved in widespread inflammatory nodules (arrowheads). Bar scale: 500μm. B) Solid line inset of Fig A. The inflammatory infiltrate comprises mononuclear cells (lymphocytes and macrophages) in both epicardium (arrows) and compact myocardium (stars). Along with the inflammation, myocardial degeneration and necrosis are also visible (arrowheads). Scale bar: 100μm. C) Dashed line inset of Fig A. Multiple foci of endo-myocarditis (arrows) and myocardionecrosis (arrowheads) in the spongy layer of the myocardium Scale bar: 100μm. D) Severe diffuse zonal degeneration and necrosis in liver. Scale bar: 250μm. E) Severe diffuse infiltrative myositis in skeletal muscle. Note the white portion of the muscle (star) completely unaffected by the inflammatory reaction. Scale bar: 500μm. F) Inset of Fig E. Severe myonecrosis (along with some regenerating muscle cells) and infiltrating mononuclear cells inflammation are evident. Scale bar: 100μm.

Histopathological results for heart and skeletal muscle evaluation in each individual fish are presented in S2 File. In nine samples out of the 469 fish evaluated by histopathology, the ventricle or even the whole heart was absent, and these samples were therefore excluded from the analysis, leading to a total of 460 fish. Lesions occurring in these samples were identified predominantly in the heart and skeletal muscle. In the heart, lesions were characterized by mononuclear cell infiltration (mainly lymphocytes and macrophages) and classified as epicarditis and myocarditis. Perivasculitis was located initially around small myocardial blood vessels, coronary veins and grooves, but with the tendency to spread with a diffuse, infiltrative pattern in severe cases. Both compact and spongy myocardium were affected in most samples, with the involvement of the latter typically in later stages of disease development, following a “centripetal” pattern (Fig 3A–3C). Lymphohistiocytic endocarditis was also present, while there was little or no involvement of the atrium. Myocardial degeneration and necrosis were also observed in association with the inflammatory pattern.

Lesions in the skeletal muscle involved red muscle fibres only. The inflammatory pattern was generally diffuse, starting from the interface between red and white muscle, and infiltrating the remaining red fibres. Some samples, usually the most extensively involved, showed evidence of fiber regeneration (Fig 3E and 3F). The lesions in the red muscle were quite inconsistent in the summer, then became highly prevalent during the autumn (56.4% in October-November 2013), primarily in the fish with the most severe lesions in the heart. They remained and were consistently present in high prevalence (75.7%) during the winter, followed by a decline in spring months, when there was evidence of the development of a recovery phase.

Secondary lesions included patchy/zonal degeneration and necrosis of the liver and/or single hepatocytes (Fig 3D), often (but not always) showing the pattern typical of heart failure. Congestion and haemorrhages in liver, kidney, spleen and gill were also occasionally present, but showed an inconsistent pattern over time. There were no inflammatory/degenerative lesions in the pancreas that might indicate the pattern associated with PD (caused by SAV), although a few samples showed extensive post-vaccination granulomatous reaction in the mesentery, eventually involving the adipose tissue around the pancreas and the pancreas itself.

**Fig 4:**
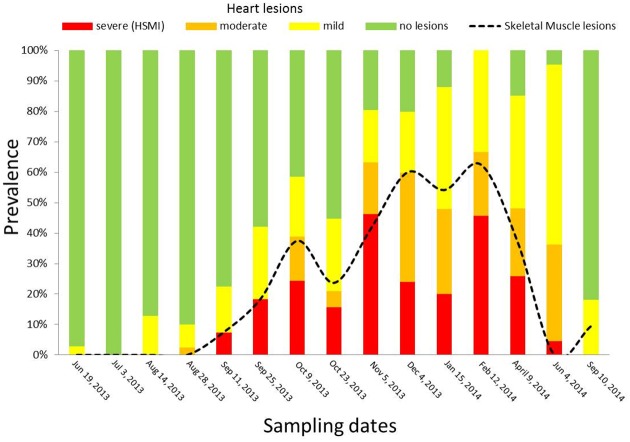
Prevalence of fish with lesions in the heart and skeletal muscle over the saltwater production cycle at the farm of interest. Bars represent the prevalence of heart lesions for sampling date. Dashed line represents prevalence of skeletal muscle lesions (skeletal muscle score ≥1). The prevalence for both tissues includes all live, moribund and freshly dead fish collected.

Following the scoring system from Finstad and colleagues [26] for heart lesions associated with HSMI in the farm of interest, a total of 75 fish out of 460 (16.3%) scored 4.0 and above, and hence were diagnosed as HSMI fish. In addition, 50 fish had scores of 3.0 to 3.5 (moderate lesions), and 97 had scores of 1.5 to 2.5 (mild lesions). These two latter groups included fish either in the developing stage or recovery stage of HSMI. The prevalence of fish showing the different degree of lesions in heart and skeletal muscle over time is shown in Fig 4. In detail, no fish showed heart lesions in the late spring/early summer 2013, the first months after saltwater transfer. A toxic algal bloom event (*Heterosigma sp*.) and mouth rot disease were observed in July 2013. In August 2013, seven fish (9.6%) showed mild heart lesions. One month later we diagnosed the first cases of HSMI, three dead fish with heart scores over four, and six additional live (clinically healthy) fish showed mild heart lesions. The peak occurrence and severity of HSMI lesions in our samples occurred 8 weeks after our initial HSMI diagnosis (November 2013), when 78% of sampled fish (including both live and moribund fish) were affected by some degree of heart lesions. A recovery stage developed by December 2013, with overall high morbidity (80% of sampled fish) and lesions characteristic of the late stage of the disease (inflammation more prominent in the spongy layer than in the compact layer of the myocardium, and milder epicarditis). However, in February 2014 there was a second peak in HSMI fish (associated with 100% morbidity of sampled fish) that lasted for two additional months, followed by a slower and longer recovery phase that persisted for 4 months, maintaining a morbidity >80%. Lastly, in September 2014, no HSMI fish were present, although four of the sampled fish (18%) still had mild lesions in the heart.

**Fig 5:**
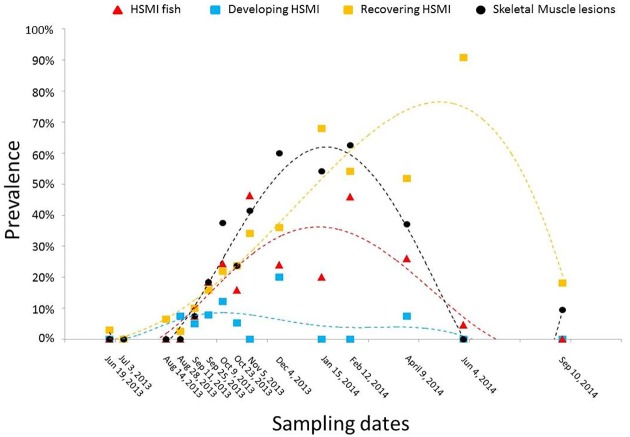
Prevalence of fish in developing stage and recovery stage of HSMI, and fish with skeletal muscle lesions over the saltwater production cycle at the farm of interest. “HSMI Fish” represents the prevalence of fish with a heart score ≥4. “Developing HSMI” represents the prevalence of fish classified in the developing stage of HSMI, with a heart score ratio (epicardium/myocardium) >1. “Recovering HSMI” represents the prevalence of fish classified in the recovery stage of HSMI, with a heart score ratio (epicardium/myocardium) ≤1.The prevalence includes all live, moribund and freshly dead fish collected. Polynomial trend lines are used to show trends on samples distribution over time for each group. Black dots (“Skeletal Muscle lesions”) and relative black dashed polynomial trend line represent prevalence of fish with skeletal muscle lesions (skeletal muscle score ≥1).

According to the further classification of fish with moderate and mild lesions based on epicardium/myocardium lesions ratio (Fig 5), the majority of the fish in the developing stage of HSMI were found in the period between late August and late October 2013 (71%). These fish displayed moderate to severe epicarditis, myocardial perivasculitis and/or mild to moderate myocarditis, especially targeting the compact myocardium, in an infiltrative pattern. On the other hand, the number of fish in the recovery stage steadily increased from a few samples in September (n = 4, 10%) to a peak of 68% and 91% among the sampled fish in January 2014 and June 2014, respectively. These fish showed milder epicarditis, while the lesions in the myocardium were still prominent in both layers, but particularly involving the spongy layer.

Based on the semi-quantitative scoring system set up for the skeletal muscle, four samples (two in September 2013 and two in November 2013) had scores of 3, 28 fish had scores of 2 and 77 fish had scores of 1. Lesions in the red fibres of skeletal muscle were first detected in September 2013 in three fish (7.5%), and became quite common by October 2013 (37.5%). Successively, the prevalence of these lesions further increased, occurring in over 50% of the samples by December 2013 and reaching 62.5% in February 2014, to eventually decline during the following spring. The overall prevalence of fish with skeletal muscle lesions is shown in Fig 4.

### Localization of *P*. *theridion* and *K*. *thyrsites* through Twort’s Gram staining

**Fig 6:**
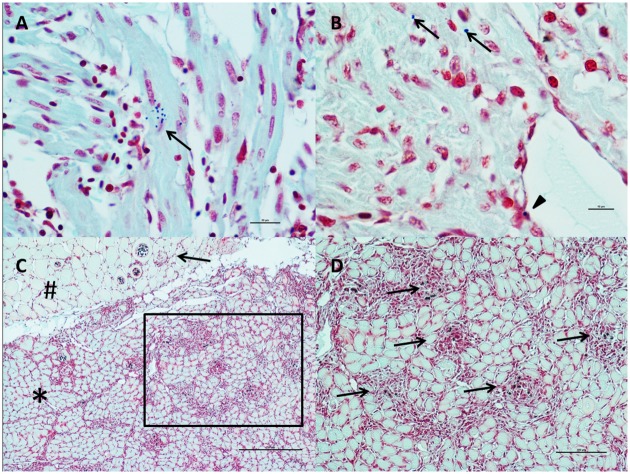
*P*. *theridion* and *K*. *thyrsites* detection in the tissues through Twort’s Gram staining. A) Cluster of *P*. *theridion* (small, dark blue organisms) inside myocardial cells in the spongy layer (arrow). Bar scale: 20μm. B) *P*. *theridion* in myocardial cells (arrows) and in an endocardial cell (arrowhead). Scale bar: 10μm. C) *K*. *thyrsites* inducing nodular granulomatous myositis in red muscle. The parasites are located in both red (star) and white (pound) portion of the skeletal muscle. One plasmodium inside a white muscle cell is also inducing a nodular granulomatous inflammatory reaction (arrow). Scale bar: 250μm. D) Inset of Fig C. Dark blue parasites are visible inside the nodular inflammation sites: 100μm.

Of the 22 fish samples stained with Twort’s Gram stain, the present study found evidence of *P*. *theridion* in gills, heart, kidney and spleen. Despite high loads of *P*. *theridion* (median Ct = 17.8, range 12.2–27.5) reported in these samples from our infective agent monitoring performed in the heart, few parasites (single cells or small clusters) were found in tissues, and none associated with any significant lesions or inflammatory reactions, but was associated with mild hyperplasia of lamellar epithelial cells in the gills of 5 samples. In the heart, the parasite was present in the myocardial cells, but with no associated reaction (Fig 6A and 6B). Moreover, there was no correlation between the load of *P*. *theridion* and HSMI, and when the parasite was the only agent present in the heart, even at high load, no fish displayed HSMI lesions.

Twort’s Gram staining also allowed the visualization of *K*. *thyrsites* in the tissues, providing a further measure to distinguish lesions due to the inflammatory reaction induced by the parasite (and by the rupture of the plasmodia) from the lesions due to HSMI, particularly in the skeletal muscle (Fig 6C and 6D).

### Immunohistochemical detection of PRV and *K*.*thyrsites*

**Fig 7:**
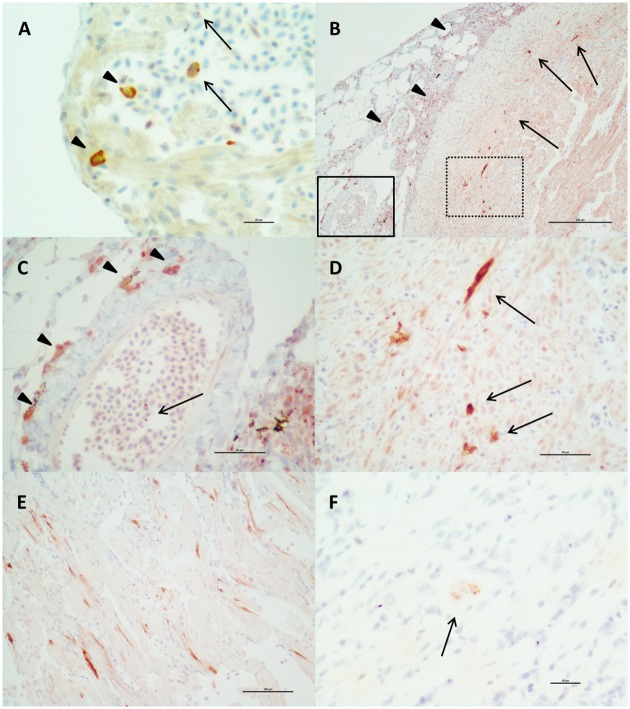
PRV and *K*. *thyrsites* detection on the tissues through immunohistochemistry. A) Red blood cells (RBCs) positive to PRV in a sample showing high PRV load but no heart lesions. The positive marking (brown—DAB) shows as intracytoplasmatic granules (tentatively called “viral factories”, arrow) or involves the whole cytoplasm (arrowheads). Bar scale: 20μm. B) PRV localization in a HSMI fish. The virus is present (red—Novared) in RBCs and leukocyte-like cells in the inflammatory infiltrate of the epicardium (arrowheads) as well as in myocardiocytes of the compact layer of the myocardium just on the border of the myocardial inflammatory infiltrate (arrows). Scale bar: 250μm. C) Solid line inset of Fig B. Leukocyte-like cells (arrowheads) and intracytoplasmatic inclusions in RBCs (arrow) positive to PRV (red—Novared). Bar scale 50 μm. D) Dashed line inset of Fig B. Cardiomyocytes of the compact layer of the myocardium, E) This sample also showed several positive cardiomyocytes to PRV (red—Novared) in the spongy layer of the myocardium. Scale bar: 100μm. F) *K*. *thyrsites* localization in the heart. Small plasmodium (red—Novared) in the spongy layer of the myocardium, in absence of inflammatory reaction. Scale bar: 20μm.

The presence of PRV antigen in heart tissues was demonstrated by immunohistochemistry. Using anti PRV Sigma-1 antibody, a strong, specific marking was observed in red blood cells (RBCs), leucocyte-like cells (tentatively identified as macrophages) and cardiomyocytes. The IHC marking occurring in the RBCs involved either the whole cytoplasm of the cells or was represented by positive granules in the cytoplasm, tentatively identified as viral factories (Fig 7A). Positive RBCs and leucocyte-like cells were demonstrated in the blood and in the inflammatory infiltrates in the heart of fish in the developing stage of HSMI (showing low degree lesions in the heart; n = 4) as well as in some fish with more severe lesions, sampled during the peak of HSMI (Fig 7B and 7C). Moreover, samples without HSMI lesions but high PRV load in the heart (n = 7; median Ct = 11.4: range 10.6–24.7) showed positive marking in the RBCs. By contrast, no positive RBCs and leukocyte-like cells were found at the later stages of the disease.

Specific staining for PRV was detected also in cardiomyocytes observed in both the compact and the spongy layer of the myocardium. Similar to the positive RBCs, the PRV-specific staining of cardiomyocytes was characterized as either an even cytoplasmic staining or as granular-like structures (Fig 7E). These results were confirmed using both anti PRV Sigma-1 and anti PRV Mu1c antibodies. The positive cells were localized within the inflamed areas on the myocardium and/or (more often) on the periphery of the inflammatory infiltrate, particularly in the samples collected during the peak of HSMI (n = 40) (Fig 7B and 7D). However, not all the HSMI fish tested showed the same degree of positivity, as samples (n = 14) showing low PRV load tested negative to the anti PRV Sigma-1 antibody, despite the presence of lesions. This observation was particularly common in late winter 2013 /early spring 2014, when the lesions were still present with a steadily decreasing PRV load (from Ct = 16.3 to Ct = 19.4).

Due to the presence of other tissues concurrently to the heart in the slides undergoing immunohistochemical staining, specific positivity for PRV with anti PRV Sigma-1 antibody was observed also in the brain (saccus vasculosus and innermost layer of cells lining the third ventricle) (S4 File), gills (Chloride cells), intestine (enterocytes) and spleen (macrophages and melanomacrophages).

Anti- *K*. *thyrsites* antibody confirmed the presence of plasmodia in cardiac, ocular and skeletal muscle (both red and white fibres) observed by H&E and Twort’s Gram staining, but allowed the visualization of additional small parasite plasmodia in the heart, that were not identified by the above mentioned staining methods. These small plasmodia were present in only two fish with very high *K*. *thyrsites* loads (Ct < 10). In the heart, *K*. *thyrsites* plasmodia were localized in the myocardium, primarily in the spongy layer, with no associated inflammatory lesions (Fig 7F). It should be noted that in three fish whereby molecular tests did not detect PRV, but detected high loads of *K*. *thyrsites* in the heart, there was no immunohistochemical marking in the heart for either PRV antibody, but a strong, specific positive marking of *K*. *thyrsites* plasmodia in the skeletal muscle was observed. These results suggest the potential for cross-reactivity of the antibodies to the parasite, although the possibility of PRV co-existing with *K*. *thyrsites* could not be completely ruled out, and further analysis must be carried out to completely understand whether a relationship between the virus and the parasite exists.

### Prevalence and load of infective agents through microfluidics-based qPCR

**Fig 8:**
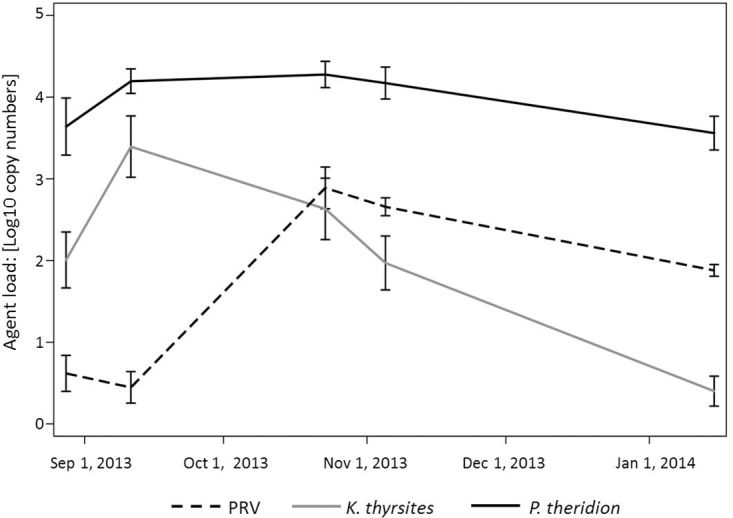
Mean pathogen load during the period (August 28^th^, 2013 –January 15^th^, 2014). Line plots of mean pathogens loads in heart tissue over time (n = 38–42), as determined by qPCR method from the BioMark^™^ platform. Data are presented as copy numbers +1, calculated based on a standard curve and normalized into a logarithmic scale base 10. Error bars represent the standard error of the mean.

Among the 44 agents tested with Fluidigm BioMark^™^ technology, only three (PRV, *K*. *thyrsites* and *P*. *theridion*) demonstrated prevalence higher that 5% in samples collected at the farm affected by HSMI, and thus considered in further statistical analysis. *Facilispora margolisi*, *Piscirickettsia salmonis* and *Parvicapsula pseudobranchicola* were also found at lower prevalence (<5%) in the heart samples. A shift in prevalence and load of PRV was observed in the autumn/winter period, when PRV infection peaked at 100% of sampled fish. PRV load increased concurrently with prevalence on the farm, coinciding with the peak of HSMI lesions. The temporal shift in prevalence of the other two parasites varied considerably. *P*. *theridion* was ubiquitous (prevalence higher that 95% for each sampling date) and with a consistent load (Ct = 19.5). On the other hand, *K*. *thyrsites* was present in this farm in about 55% (n = 22) of the fish in summer 2013, increasing in prevalence and load in early autumn, and eventually decreasing through the colder months (Fig 8).

It is noteworthy to mention that all the heart tissues tested in this study (n = 188 out of 190, as two samples with no housekeeping gene expressions must be excluded) were negative to the other two viruses commonly related to heart lesions in Atlantic Salmon in Europe (i.e. SAV and PMCV), causal agents of PD and CMS, respectively.

### PRV genome sequence analysis

Next-generation sequencing (NGS) was utilized to verify the detection of PRV in two of the analysed Atlantic Salmon heart tissues, B5690 (PRV Ct of 10.6) and B7274 (PRV Ct of 12.1). The first sample (B5690) was identified as an HSMI fish (heart score of 6 and skeletal muscle score of 3) and came from a dead fish collected on September 11, 2013 while the second sample (B7274) was identified as HSMI negative (heart and skeletal muscle scores of 0) and came from a live fish collected on Oct 23, 2013. These two samples were processed on separate Illumina MiSeq runs and generated 9,050,063 and 6,249,127 post trim reads, with average quality scores of 35.69 and 35.94, respectively.

The HSMI sample (B5690) generated 52,647 total alignments (0.3% of total reads) to the Norwegian PRV Salmo/GP-2010/NOR 10 segment reference genome [25] which resulted in 100% coverage with an average depth of 326,62 reads (30X coverage for 98% of the genome). Segment L3 (core RdRp protein) displayed the least variation (0.5%/18 SNPs) and segments M2 (outer shell protein) and S1 (outer clamp protein) displayed the most (3.1%/67 and 34 SNPs, respectively). Blast searches revealed that our B5690 PRV isolate was identical at segments S1 (outer clamp protein), S3 (non-structural RNA protein) and S4 (outer fiber protein) to previously published genomes from BC, Genbank accession numbers KT456503, KT429758, and KC795576, respectively. Of the 10 PRV segments, 7 were most homologous to those isolated from farmed Atlantic Salmon (Canada, 2012 to 2014), while 3 (L2, L3 and S3) were most homologous to those isolated from wild Coho (Columbia River, U.S., March 2014).

The PRV positive/HSMI negative sample (B7274) generated 4,172 alignments (0.03% total reads) when aligned to the PRV B5690 reference genome which resulted in 98% coverage with an average depth of 30 reads (30X coverage for 36% of the genome). This sample was approximately 99.9% similar to this PRV positive/HSMI positive sample over all segments. Segment L1 (core shell protein) displayed the greatest variation between the two PRV isolates (0.2%/8 SNPS), while segment S1 (outer clamp protein) was the only segment that was identical between the two. Phylogenetic analysis of segment S1 revealed that both PRV isolates in this study grouped into the sub-genotype Ia, which contains all of the Canadian PRV strains reported to date (S5 File). Our S1 segment sequence was most divergent (16.9%/181-183 SNPs) from the recent Chilean farmed Coho Genbank deposits (Genbank accession numbers KU131595 and KU131596) that have been designated into a new genotype group (Genotype II) [6].

Finally, as in the infective agent monitoring, neither of these samples generated aligned reads to the other two viruses (i.e. SAV and PMCV) commonly related to heart lesions in Atlantic Salmon in Europe.

### Statistical association between pathology and infective agent

**Fig 9:**
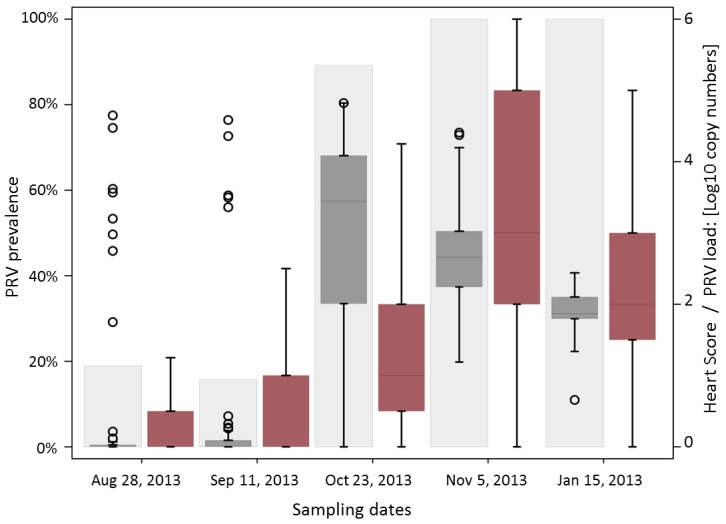
Prevalence and load of PRV in association with heart lesions. Plot of PRV prevalence (light grey plots) and load (dark grey box plots) in the heart over time. PRV load data are presented as copy numbers +1 calculated based on a standard curve and normalized into a base 10 logarithmic scale, ranging between 0 and 6. Black circle points define outliers. Heart score (red box plots) corresponds to the sum of the scores for both myocardial lesions and epicardial lesions, rated between 0 and 3 according to the severity (total score ranging between 0 and 6).

All failed samples for histopathological evaluation included in the five sampling dates tested through the agent molecular screening (n = 5) were excluded from the statistical analysis. The same conditions applied to seven failed samples for molecular testing (two fish showed no housekeeping gene expression, five samples showed single Ct value for PRV out of the two duplicates), leading to a total number of 178 samples undergoing statistical analysis. All fish diagnosed with HSMI and tested with Fluidigm BioMark^™^ technology tested positive for PRV infection in the heart (n = 33), mostly at high load. Moreover, all fish with a heart score of 3.0 or 3.5 were positive for PRV (n = 17) as well as most of the fish with scores between 1.5 and 2.5 (n = 30 out of 35). On the other hand, not all fish with high PRV loads exhibited histopathological signs of HSMI. Overall, the presence of PRV in heart tissue was associated with the presence of heart lesions (chi-square test, p<0.001) while there was no apparent association between the presence of *K*. *thyrsites* or *P*. *theridion* and occurrence of lesions (p = 0.240 and 0.877, respectively). Also, the severity of the lesions was associated with the presence of PRV, where the higher the heart score, the higher prevalence of PRV detection in heart tissue (Fig 9). None of the interaction terms tested were statistically significant. Our final ordinal logistic regression model for heart scores included the five sampling dates (p<0.001), fish condition (D, M, or L; p = 0.003), and PRV status (p = 0.001) from 178 fish between August 2013 and January 2014 (S1 Table).

**Fig 10:**
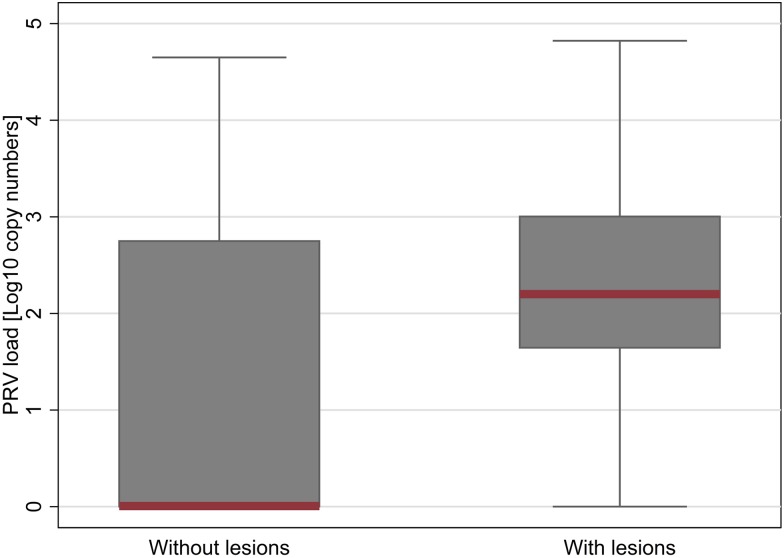
PRV load in association with heart lesions. Box plots of PRV load in heart tissue of fish analysed on the basis of the presence of heart lesions. Median of PRV load is highlighted in red. PRV is presented as copy numbers + 1 normalized into a base 10 logarithmic scale. Presence of lesions is defined by a histopathological score in myocardium and epicardium equal or higher than 1 in a scale of 0–6.

Overall, there was a significant association between the PRV status and histology scores, where PRV-positive fish increased the odds of being at or above any given heart score category compared with being below that score category by 8.9 times. In other words, the odds were approximately 9 times greater for PRV-positive fish to have higher heart scores compared to PRV-negative fish (p = 0.001; S1 Table) (Fig 10). Those samples collected from dead fish were 5.7 times more likely to have higher heart scores than live fish (p = 0.001), while we did not observe any explicit differences between moribund and live fish (OR 1.1; p = 0.861) (S1 Table). Quantitatively, the load of PRV in heart tissue was significantly higher for fish with lesions compared with fish without lesions (p<0.001; analysed with the non-parametric Wilcoxon rank-sum test, while that wasn’t the case for the two parasites *K*. *thyrsites* and *P*. *theridion* (p = 0.505 and 0.273, respectively).

## Discussion

Through intensive analyses of both live, moribund and recently dead fish over the entire production cycle of one farm, our study documented the development, peak and recovery phases of HSMI occurring over an extended duration, in a manner that was consistent temporally and pathologically with findings of HSMI in Norway, as reported in the longitudinal study by Kongtorp et al. [11].

Lesions diagnostic of HSMI (according to Biering and Garseth [7]) at various degrees of severity were reported for an 11-month timespan. In the heart, lesions first appeared 6 weeks after a harmful algal bloom event and ensuing development of mouth rot disease on the farm. These initial lesions preceded by 1 month the first fish diagnosed with HSMI (heart score ≥4), while the peak of the HSMI event occurred 2 months later (7 months post sea transfer). The following two months showed a decrease in number of HSMI fish but, unlike Kongtorp’s longitudinal study, by February 2014 a second spike in HSMI fish occurred, coinciding with a high number of sea lice and corresponding delousing treatment. Handling procedures and different types of stressors have been identified in Norway as potential risk factors for the disease [9,18,23]; therefore we speculate that the occurrence of this disease could have been triggered by the harmful algal bloom event (creating hypoxic conditions) that occurred in the late summer 2013, and that handling associated with the delousing treatment may have further compromised the fish, bringing about new HSMI cases in February 2014. However, because fish were collected from different pens at each sampling date, we can’t exclude the possibility that fluctuations in severity of histopathological lesions reported in these samples could be a sampling artefact; but we can conclude that the study documents a farm-level case of HSMI.

The high farm-wide prevalence of inflammatory lesions in the heart reported in our study is also consistent with HSMI, which in Norway can reach up to 100% of the farm population, even when mortality is low or negligible [9,11]. Indeed, more than 80% of the sampled fish had lesions consistent with HSMI across multiple pens for a span of 7 months, from November 2013 to June 2014, including live-sampled and dying fish.

While our study did not undertake a systematic approach to the notation of gross clinical signs, observations on moribund and recently dead fish included abnormal behavior (“fish off feed”) and the presence of macroscopic lesions, such as ascites, pale heart, brain congestion, inflammation and haemorrhages, congestion of liver and spleen associated with circulatory disturbance, that are consistent with findings reported for HSMI in Norway [9], although not specific (i.e. pathognomonic) of the disease. In particular, ascites, common outcome of heart failure, was the gross lesion most strongly tracking the development of HSMI, affecting over 40% of fish during the peak of HSMI in October-November 2013. Gross signs of brain inflammation showed the same HSMI-related pattern, although no lesions were reported microscopically. This represents the first report of brain lesions concurrently found with HSMI, but given discordance between gross and microscopic evidences, should be further evaluated. Unusual mortality and clinical signs were also noted on two laboratory submissions from the farm veterinarian sent for further analysis and coinciding with the peak of HSMI fish in October-November 2013; mortality data suggest that farm-wide mortality during that period was not unusually high, but there was slightly increased mortalities affecting predominantly three out of twelve pens. In Norway, HSMI does not always result in elevated mortality (range 0–20% [1]), production impacts [9], or even clinical signs of disease [11]; and in fact, laboratory challenge studies with HSMI positive tissues have emulated the lesions, but not the mortality or clinical signs [17,27].

The pattern and distribution of histopathological lesions occurring in heart and skeletal muscle described in our study are consistent with the findings reported in Kongtorp’s longitudinal study. In addition, our study demonstrated the temporal shift in presence, severity and distribution of lesions in heart tissue, providing a fine-scale resolution of disease development at the farm level. The vast majority of fish in the developing stage of HSMI were reported in the period between August and October 2013, indicating the progression of the lesions from outside (epicarditis and perivasculitis) to inside of the heart tissue, spreading to the myocardium and inducing a myocarditis involving principally the compact layer. In November 2013, HSMI fish showed moderate to severe lesions occurring in both epicardium and myocardium. At this point, both compact and spongy layers of the myocardium were involved, although the compact layer was still more affected. Just after the peak in occurrence of fish diagnosed with HSMI, we recorded a substantial decrease in the proportion of fish in developing stage HSMI, while the number of fish in the recovery stage of HSMI steadily increased, reaching a first peak in January 2014. In this time frame, lesions were generally milder, in particular in the epicardium and compact myocardium, while they remained quite consistent in the spongy layer of the myocardium, demonstrating the end point of the “centripetal pattern” of HSMI development. Interestingly, no fish in the developing stage of HSMI were reported concurrently to the second peak of HSMI occurring later in February 2014, indicating that these HSMI cases might more likely represent a deterioration of the pre-existing recovery condition rather than a recrudescence of the disease. As previously mentioned, we cannot exclude that this finding is an artefact due to sampling from different pens along the study, but the presence of a second peak of recovery stage fish in June 2014 might suggest that the second spike of HSMI fish in February 2014 was real. In September 2014 (the last sampling date available for this study), no HSMI fish were detected, although there still remained some mild to moderate lesions principally in heart and liver tissues, suggesting the slow pace of recovery of affected fish surviving HSMI.

Lesions in the skeletal muscle usually started from the interface between red and white muscle, and tended to infiltrate the remaining red fibres, a pattern that was easily differentiated from the nodular/granulomatous lesions induced by *K*. *thyrsites*. Overall, these lesions were quite inconsistent in summer 2013, before HSMI was evident, but became highly prevalent during autumn 2013, primarily in the fish with the most severe lesions in the heart, while they were less common and milder during winter and spring 2014, as the recovery phase developed. This finding is also consistent with Kongtorp’s longitudinal study and others [10,11], confirming the necessity for the presence of these lesions at a farm level to diagnose HSMI and differentiate it from CMS, but not at the individual level.

Negative molecular tests for SAV and PMCV along with the absence of typical lesions reported for PD (pancreas involvement) and CMS (epi-myocarditis, but only atrium and spongy layer of the myocardium typically affected, and no skeletal muscle involvement) further strengthened the final diagnosis.The two diseases might be found mixed with HSMI, making the diagnostic pathway more challenging in that region [24]. Moreover, the absence of these particular concomitant infections in BC salmon might help explain why mortality was low.

### Correlation between PRV and HSMI

A typical element of reported HSMI cases in Atlantic Salmon has been the concurrent presence of PRV [17,18,25]. Despite the fact that, to date, HSMI has never been reproduced in the laboratory or reported at a farm-level in the absence of PRV [15,16], a causal relationship between PRV and HSMI has not been demonstrated. Two recent challenge studies on BC salmon concluded that they were unable to replicate HSMI or any evidence of disease or mortality in Atlantic Salmon from PRV-positive inoculum from healthy Atlantic Salmon [34] or from tissues associated with jaundice Chinook salmon (*Onchorynchus tshawytscha*) which also contained PRV [35]. A low pathogenic strain of PRV in BC, or lack of a causative relationship between PRV and HSMI were hypothesized to account for these results. These studies demonstrated the transmission of PRV via intra-peritoneal (IP) injection [34,35] and cohabitation [34], and its active replication in Atlantic Salmon. Mild heart lesions (lymphohistiocytic endocarditis and/or epicarditis) were reported in a few, mostly naïve, fish cohabiting with PRV IP-injected fish in the most recent study [34], and approximately 20% of challenge fish (but no control fish), in the other study [35].

Our farm-level study was not designed to investigate causal relationships between any agent and histopathologic lesions. However, our analytical evaluation of longitudinal samples demonstrated a statistically significant correlation between PRV prevalence and load with the occurrence and severity of HSMI lesions in the heart, a correlation that had previously been demonstrated in laboratory-based challenges [17,25] as well as in the field [18]. Setting this study apart from all others performed to date, we conducted both a broad surveillance of known microbial agents, virtually all with pathogenic potential in salmon, and follow-up RNA-seq on two affected fish to obtain a full genome sequence of PRV. We demonstrated that of the agents tested in the heart samples, PRV was the only agent detected in heart tissue that was correlated with HSMI lesions in the heart. *P*. *theridion* was consistently present in nearly all of the farm population, at high load in some individuals, but without statistical correlation with live-sampled and moribund/dead fish or with HSMI lesions in heart tissues. The other common agent, *K*. *thyrsites*, was observed as a co-infection most notably in the summer months, decreasing around the time that the first HSMI fish were observed. Using IHC, we demonstrated the presence of *K*. *thyrsites* in heart and skeletal muscle, but no inflammatory reaction was detected in the heart and was different (multi-focal/granulomatous type) and straightforward to distinguish from the diffuse, infiltrative pattern induced by HSMI in the muscle, in which PRV in the heart tissue was commonly observed. In our study, along with the demonstration that all fish with a heart score ≥ 3 were positive for PRV (and most with scores between 1.5 and 2.5), the odds for PRV-positive fish to have higher heart scores compared to PRV-negative fish were approximately 9:1. In addition, PRV load was significantly higher in fish with heart lesions than in fish without lesions, as also demonstrated by Løvoll et al. [18].

Along with the molecular evidence showing a statistical correlation between PRV replication and developing HSMI lesions, our IHC evaluation localized PRV to the areas of HSMI-associated inflammation and within the cardiomyocytes, findings consistent with IHC from laboratory-based challenges [26], but reported for the first time from a field setting. We found that early in the development of the disease, in fish with high load of PRV without evidence of inflammatory lesions in the heart, the virus was distributed strictly in the RBCs, consistent with the findings of Finstad et al. [26] who demonstrated that erythrocytes are the primary infective cells for PRV. During the peak of the disease, PRV loads were generally high and the virus was mostly localized in the cardiomyocytes within the inflamed areas of the myocardium and/or (more often) on the periphery of the inflammatory infiltrate. This localization of the virus could be associated with the phase of cell-mediated response of the organism that is expressed by a typical mechanism of a Type IV hypersensitivity reaction (characterized by CD8+ lymphocytes and macrophages, as described for HSMI by Mikalsen et al. [17]). If PRV were to have a role in the development of HSMI, it is possible that the virus may replicate and be tolerated in long-lived erythrocytes moving through the circulatory system, but when the virus is released due to stress or other factors (such as hypoxia), it could pass to the cardiomyocytes, inducing degeneration of the cells and activating an autoimmune reaction caused by the release of myocardial myosin. This mechanism would be consistent with the action of myocarditic reoviruses in other species [36,37], as well as several other viruses inducing myocarditis [38]. During the recovery phase of HSMI, when farm-level PRV loads were generally reduced, we had more difficulties visualizing PRV in individual fish, as others have reported [26], despite the continued presence of lesions. Finstad and colleagues [26] suggested that if PRV plays a role in the development of HSMI, the immune system could clear the virus more quickly and effectively than the time it takes to repair the damage induced by inflammation, an observation that could also explain why occasionally fish with HSMI lesions can have lower PRV loads than apparently healthy fish, or even no PRV at all.

Another causal hypothesis is that, given the relatively high prevalence of this virus in farmed salmon, background PRV infection may be one of the few agents detected when health is compromised by other extraneous factors [19]. Alternatively, compromised health from any source may provide the opportune host for PRV proliferation [34]. In this case, PRV may simply represent a component cause of HSMI, rather than a necessary cause of HSMI. While intriguing, this hypothesis does not explain the changes in PRV concentration in erythrocytes predominantly seen in the early stages of HSMI to cardiomyocytes in the peak of the disease at an individual level nor the specific spatial localization in tissues between the virus-infected cardiomyocytes and inflammatory lesions.

PRV was localized (though immunohistochemnistry with anti PRV Sigma-1 antibody) in tissues additional to the heart. As PRV primarily infects RBCs, the virus should be present across multiple organs; however, the localization of the virus within tissues other than RBCs was quite cell-specific. In the brain, PRV was detected primarily in cells of the saccus vasculosus, a sac-like structure in strict contact with the blood, and the inner layer of the cells covering the third ventricle receiving the cerebrospinal fluid produced by the saccus vasculosus. The enterocytes also showed positivity to the antibody, confirming the finding of Hauge et al. [39], who demonstrated a transmission of PRV (and HSMI) through anal administration of PRV. In the spleen, positivity was primarily found in macrophages and melanomacrophages; we cannot distinguish whether these cells were positive because they were phagocyting virus particles or damaged infected RBCs. Finally, the presence of PRV in the gills might represent a possible transmission route for either viral infection or shedding. These findings could add information regarding the PRV infection in other organs and cell types than heart and erythrocytes; however, confirmative studies are required to fully understand the pathogenesis of the infection.

### HSMI in BC

In the regulatory disease surveillance program for farmed BC salmon (the DFO Audit program), farms to be sampled are chosen at random on a quarterly basis, not in response to mortality events, although they may occasionally be captured, with only a small number of fresh silver fish (recently dead; generally 2–6) assessed per farm on a given sampling event. While myocarditis lesions have been reported within this program sporadically across multiple BC farms since 2002, no specific disease, including HSMI, has been diagnosed in association with these lesions. The case definition applied to diagnose HSMI by BC veterinarians required the presence of inflammatory lesions in both the heart and concurrent skeletal muscle lesions in individual fish (where skeletal muscle tissue was provided), and accompanying clinical signs and increased mortality at the farm level [40,41].

Our study focussed intensive analysis on one farm in the region where these lesions were observed across four regulatory farm audits from 2011–2013 (S1 File). Using the international standard of specific pathological lesions in heart and skeletal tissues as the case definition for HSMI, we diagnosed HSMI at both the farm and individual level occurring from seven months post seawater entry until close to harvest 11 months later. HSMI is one of three viral heart diseases in Norway, and we were able to definitively rule-out the other two diseases (CMS and PD) because of lack of detection of the causative agents and through the differential tissue distribution of the lesions. As a result, it is conceivable that some of the occasionally observed myocarditis lesions on farms in BC dating back as far as 2002 may have been HSMI.

There are two plausible explanations why the disease HSMI was diagnosed in this longitudinal study, but not previously. Firstly, our study focussed intensive temporal sampling of live and dying salmon on one farm in the region where heart lesions have most commonly occurred (S1 File) and was able to capture the full development of the disease over hundreds of fish sampled. We showed that, at very early and late stages of the disease, the linkage between heart and skeletal muscle lesions within individual fish could be relatively weak. The inconsistency between heart and skeletal muscle pathology at early and late stages of HSMI has also been observed in farm outbreaks [11] and laboratory challenges [1] in Norway. Hence, skeletal muscle pathology is an important differential for the diagnosis of HSMI at the farm level, but it is not an appropriate differential at the individual fish level. However, in our study, at the peak of the disease, a large proportion of fish were affected with severe heart and skeletal muscle lesions, as would be expected; as such, our data provide high confidence in the diagnosis of HSMI on this farm. Alternately, in sampling programs that may capture only a small number of fish on a farm, whether in response to a mortality event or as in the Audit program, as a random sampling event at non-peak stages of disease, it may be difficult to diagnose HSMI with a high degree of confidence. Moreover, if the disease is not associated with elevated mortality, or if clinical signs are missing, the farm would be unlikely to conduct diagnostic testing on their fish, and thus may not capture the disease, or may observe the lesions at a time when there is a background of other common infections.

Secondly, the disease would likely be missed under case definitions that require elements other than histopathological lesions in HSMI diagnosis, again especially if small sample sizes are used. Our study did document some clinical evidence consistent with HSMI outbreaks in Norway, with the occurrence of clinical signs maximized during the peak of the outbreak. However, previous studies have shown that fish with HSMI do not necessarily all have the same clinical signs, and many appear healthy with no clinical signs, even with high lesion scores [11]. For this reason, clinical signs are not a differential for HSMI in Norway. In our study, there were no unusually high numbers of mortalities coinciding with HSMI fish at the farm-level, as observed between September 2013 and February 2014, although up to three pens showed slightly increased mortality in that timeframe. However, because in Norway HSMI is not always associated with elevated mortality, this parameter is not used as a differential for the disease. Finally, we showed that PRV is statistically associated with the development of heart lesions, but also that some fish carry PRV without lesions, especially early in the disease, as has also been reported in Norway [20,26], and we were further able to resolve through immunohistochemistry that in these fish, PRV appears to be confined to red blood cells rather than cardiac cells. We showed as well that while PRV was always detected in HSMI fish (heart score ≥ 4), and mostly at high load, PRV was detected in fairly low load in some recovering fish; this is consistent with other studies [26] and is one reason (in addition to the lack of a confirmed causative relationship) that PRV is not used as a differential in the diagnosis of HSMI in Norway, but is often used in a confirmatory role (Dr. Torunn Taksdal, Personal Communication).

### Wild Pacific salmon

In BC, the farmed Atlantic Salmon industry shares waters with large and abundant wild Pacific salmon stocks, and there is a strong interest in ensuring that the salmon aquaculture industry is sustainable in an environment with pathogen exchange potential between wild and farmed salmon. Policy decisions require strong scientifically-defensible data to support this overall objective [42]. The longitudinal farm study reported herein was motivated by these information needs, specifically addressing whether the historically reported heart lesions on the targeted farm and in that Aquaculture Management Area could, in fact, derive from the emerging disease HSMI. Our data clearly show that the pathological lesions diagnostic for this disease were broadly present within the Atlantic Salmon on this single farm, and that the only known virus to routinely associate with the disease is also associated with HSMI lesions on the farm.

The obvious next question becomes, what is the risk of this disease in Pacific salmon, and/or the risk of transmission of this virus between wild salmon and farmed salmon? Risk assessment will require further studies, but PRV has been detected in most Pacific salmon species that have been tested in BC, Washington and Alaska, at lower prevalence than on farms (0–21% vs >70%) [19,43,44]. Although HSMI has never been diagnosed in any Pacific salmon species in BC [19] or in wild Atlantic Salmon in Norway [20,21] to date, lack of access to representative samples throughout the life cycle limits the conclusions possible for the natural history of the complex relationship between PRV and HSMI in wild fish populations. However, HSMI-like lesions have been reported concurrent with anemia and necrotic lesions in the kidney and liver (often resulting in a jaundice appearance in the fish) in farmed *Oncorhynchus* species in Norway (*Oncorhynchus mykiss*) [45] and Chile (*Oncorhynchus kisutch*) [6]. In both farm studies, PRV was also observed, but whether this was due to its high prevalence on farms in general, or to a role in the development of the disease is unknown. Jaundice and anemia has also been reported in farmed BC Chinook salmon, *O*. *tshawytscha*, coincident with PRV infection, but a challenge study failed to reproduce mortality, clinical signs, or the lesions found on fish dying of jaundice on the farms [35]. The study did, however, identify mild lymphohistiocytic endocarditis lesions exclusive to challenged fish and in all the three species tested (Chinook [60%], Sockeye [70%] and Atlantic [20%] salmon), but none of these fish carried lesions in the skeletal muscle. Just as in Atlantic Salmon, future studies are required to address whether there is a causal relationship between PRV and disease in any salmon species.

## Conclusions

In summary, our longitudinal study identified HSMI over an extended period in 2013–2014 on one farm in BC, Canada, and documented the same correlation between PRV and HSMI lesions in heart tissues as reported for HSMI occurrences in Norway. This is the third country in which PRV has been associated with HSMI lesions, providing supporting evidence that PRV appears to be a component for HSMI development, but falling short of demonstrating that PRV alone is sufficient to cause HSMI. However, it is important to put these data into perspective given what we do not know about the role that PRV has on HSMI in BC salmon. Our data describe the occurrence of HSMI lesions on one of four Atlantic Salmon farms we followed through an entire ocean production cycle. Hence with these data alone, we cannot comment on the spatial extent of this disease or potential impacts on other species, such as wild Pacific salmon. Future epidemiological studies need to be extended both geographically and temporally, to identify the extent of the disease, to further evaluate the relationship with PRV, and to further elucidate predisposing factors that may contribute to the development of HSMI in the field, including environmental and husbandry practices, and strain variation derived through full viral genome sequencing. In addition, challenge studies should be conducted using HSMI positive tissues in both Atlantic and Pacific salmon to assess the disease potential across species and the contribution of PRV (or other identified agents) and additional contributing factors to the development of HSMI lesions.

## Supporting information

S1 File Data extrapolated from the Department of Fisheries and Oceans—Aquaculture Management Division—Fish Health and Surveillance Audit program. The data show heart lesions identified on four farms located in the region of the farm of interest, for years 2011–2013.
(XLSX)

S2 File Complete sample inventory detailing the fish number, sampling date, associated metrics, histopathological and molecular results, and analysis carried out for each fish collected. (XLSX)

S3 File List of all the infective agents tested through Fluidigm BioMark^™^ microfluidics-based qPCR system. (XLSX)

S4 File Immunohistochemical detection of PRV using PRV Sigma-1 antibody on the brain. A) Interstitial cells of the saccus vasculosus (arrows) show presence of PRV (red—Novared). Bar scale 50 μm. B) PRV is also present in the innermost layer of cells lining the third ventricle (arrows) (red—Novared). Bar scale 100 μm.
(TIF)

S5 File Molecular Phylogenetic analysis of Piscine orthoreovirus segment S1 isolates from Canada, Chile, Norway and Japan by Maximum Likelihood method. Phylogenetic relationships were inferred by using the Maximum Likelihood method based on the Kimura 2-parameter model. Bootstrap analysis (1,000 replicates) was used to validate tree topology. The percentage of trees in which the associated taxa clustered together is shown next to the branches. Initial tree(s) for the heuristic search were obtained automatically by applying Neighbor-Joining and BioNJ algorithms to a matrix of pairwise distances estimated using the Maximum Composite Likelihood (MCL) approach, and then selecting the topology with superior log likelihood value. The tree is drawn to scale, with branch lengths measured in the number of substitutions per site. The analysis involved 111 nucleotide sequences and there were a total of 827 out of 1,081 nucleotides used in the analysis. The two sequences derived from this study (B5690 and B7274) are indicated with black triangles while the new divergent PRV isolate from Coho in Japan (LC145616) is indicated with a black circle.
(TIF)

S1 Table Ordinal logistic regression modeling the 3-category heart lesion score (HS0 = total heart score≤1, HS1 = total heart score >1 and <4, and HS2 = total heart score ≥4) from 178 fish (samples collected from Live, Moribund, or Dead fish) taken over five sampling events from one farm in British Columbia, Canada. Heart tissues from each fish were tested for the presence of PRV (1/0), using qPCR methods on the BioMark^™^ platform. Estimates are reported in both linear (coefficient and corresponding standard errors) and multiplicative (odds ratio with corresponding 95% confidence intervals) scales.
(TIF)
