# Supplementary figures and images for: Heart and skeletal muscle inflammation (HSMI) disease diagnosed on a British Columbia salmon farm through a longitudinal farm study
Source: PLoS One. 2017 Feb 22;12(2):e0171471. doi: 10.1371/journal.pone.0171471 (PMC5321275; doi:10.1371/journal.pone.0171471)

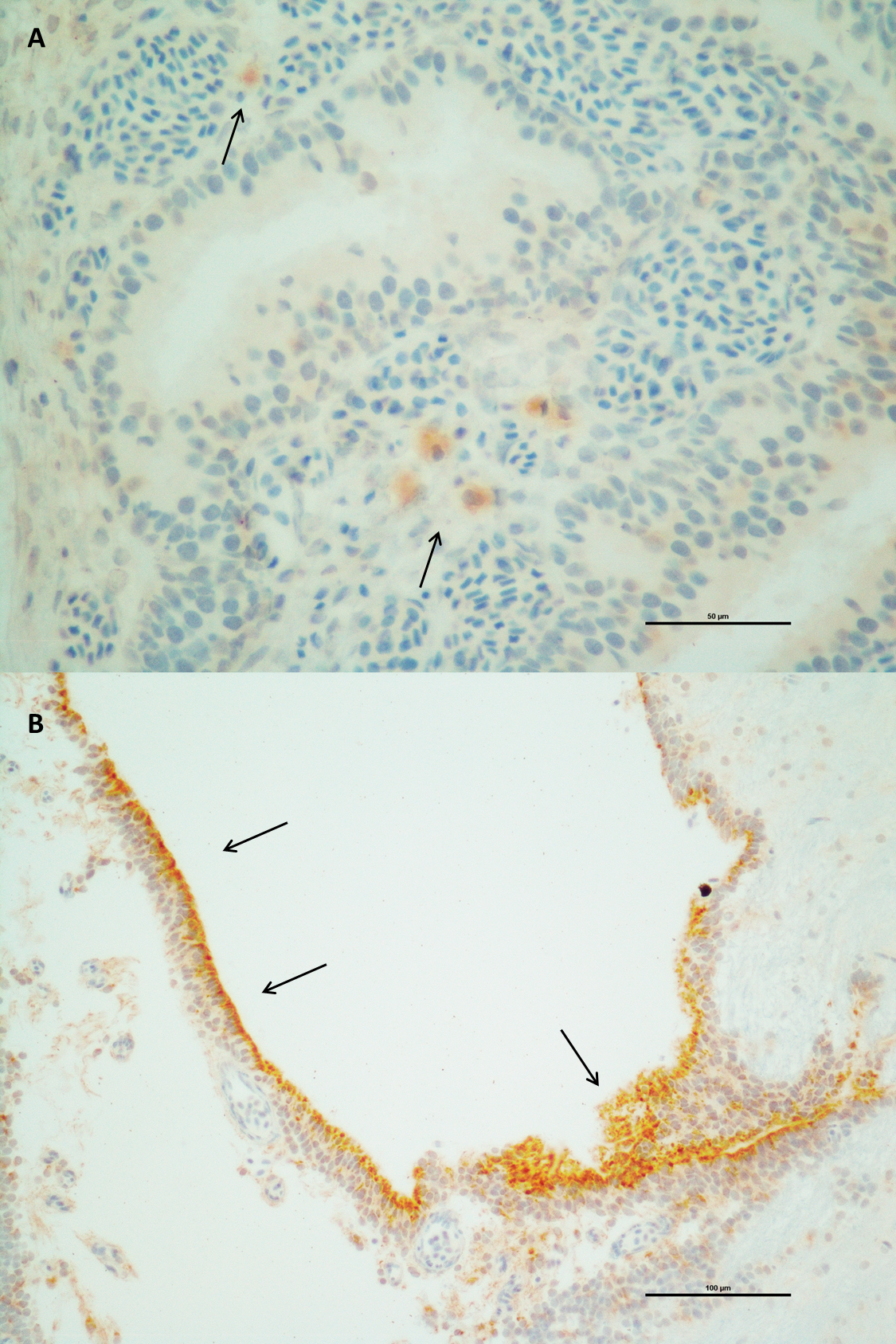

Supplement: S4 File — A) Interstitial cells of the saccus vasculosus (arrows) show presence of PRV (red—Novared). Bar scale 50 μm. B) PRV is also present in the innermost layer of cells lining the third ventricle (arrows) (red—Novared). Bar scale 100 μm. (TIF) [file pone.0171471.s004.tif]

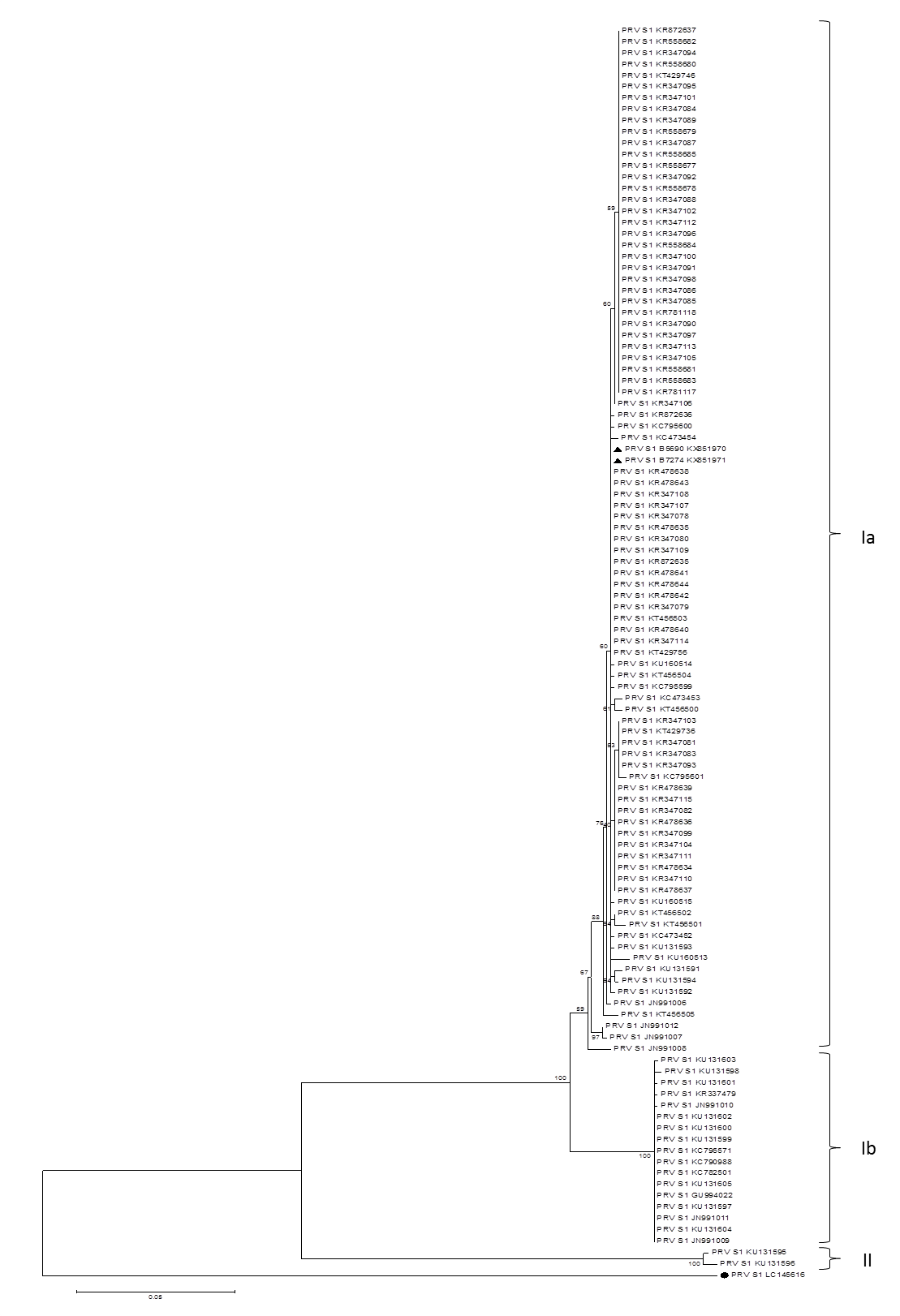

Supplement: S5 File — Phylogenetic relationships were inferred by using the Maximum Likelihood method based on the Kimura 2-parameter model. Bootstrap analysis (1,000 replicates) was used to validate tree topology. The percentage of trees in which the associated taxa clustered together is shown next to the branches. Initial tree(s) for the heuristic search were obtained automatically by applying Neighbor-Joining and BioNJ algorithms to a matrix of pairwise distances estimated using the Maximum Composite Likelihood (MCL) approach, and then selecting the topology with superior log likelihood value. The tree is drawn to scale, with branch lengths measured in the number of substitutions per site. The analysis involved 111 nucleotide sequences and there were a total of 827 out of 1,081 nucleotides used in the analysis. The two sequences derived from this study (B5690 and B7274) are indicated with black triangles while the new divergent PRV isolate from Coho in Japan (LC145616) is indicated with a black circle. (TIF) [file pone.0171471.s005.tif]

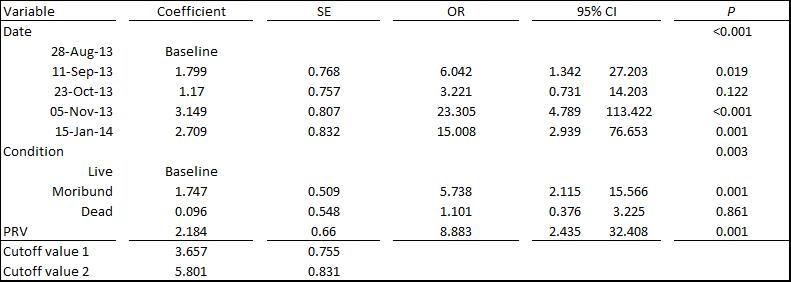

Supplement: S1 Table — Heart tissues from each fish were tested for the presence of PRV (1/0), using qPCR methods on the BioMark™ platform. Estimates are reported in both linear (coefficient and corresponding standard errors) and multiplicative (odds ratio with corresponding 95% confidence intervals) scales. (TIF) [file pone.0171471.s006.tif]
